# The effect of cultural capital on the physical fitness level of a Chinese older adult population: chain mediation of household income and stockpiling of physical fitness goods

**DOI:** 10.3389/fpubh.2024.1473775

**Published:** 2024-11-18

**Authors:** Deqiang Zhao, Yibei Wang, Aoyu Zhang, Jin He, Yibo Gao, Xiaoxiao Chen, Lupei Jiang, Yanfeng Zhang

**Affiliations:** China Institute of Sport Science, Beijing, China

**Keywords:** older people, cultural capital, physical activity, fitness level, intermediary effect

## Abstract

**Background:**

Health behaviors of older people are influenced by many factors, and physical activity are important lifestyle behaviors that promote healthy aging.

**Purpose:**

This study is to analyze the intrinsic mechanism of the influence of cultural capital on the physical fitness level of older people, and to provide a theoretical basis for the improvement of the differences in physical fitness level caused by the differences in physical fitness concepts of the classes brought about by cultural capital, and the unequal distribution of resources.

**Methods:**

The subjects of this study were derived from people over 60 years old in the 2020 China National Fitness Activity Status Survey, and a total of 20,896 samples were obtained using the principle of multi-stage stratified random sampling. The dependent variable was assessed by the Physical Activity Rating Scale (PARS-3) to calculate the physical activity level score of the older adult population. Pearson correlation analysis and stratified regression methods were used to analyze and explore the factors influencing the physical fitness level of sport older adult people, followed by quantile regression to explore the distribution of the influence of institutional cultural capital in different physical fitness levels. The use of quantile regression not only provided a robust test of the results of stratified linear regression, but also analyzed the differential effects of institutional cultural capital among individuals with different fitness levels. Finally, Bootstrap methods were used to test the mediating effects of household income and physical cultural capital.

**Results:**

Institutional cultural capital (*p* < 0.01), household income (*p* < 0.01), physical cultural capital (*p* < 0.01), and health status (*p* < 0.01) are all conducive to improving fitness levels among older people. Family income (95%CI = [0.467, 0.235]) and material cultural capital (95%CI = [0.199, 0.291]) play a chain mediating role.

**Conclusion:**

Cultural capital has a positive impact on the health of older people, mediated by household income and stocks of sporting goods. An increase in the level of cultural capital of older persons is beneficial to the improvement of health perception. Therefore, it is possible to promote the improvement of physical fitness among older people through the enhancement of their cultural capital and to realize healthy aging.

## 1 Background

Health behavior is influenced by many factors, and each factor acts comprehensively on human physical activity and health level. In the study of physical fitness behavior, many scholars are concerned with the important question of what factors influence popular physical fitness? Senectitude as a specific life stage, the decline of physical function as well as physical health disadvantages become more and more prominent (1). Physical fitness activities can widely improve physical functioning and reduce the risk of chronic diseases in older adults (2, 3). Promoting older people to maintain regular physical activity has been the focus for scholars. In social-ecological system theory, it believes that human behavior is influenced by factors in the individual’s internal environment (e.g., the individual’s motivation, beliefs, etc.) and factors in the individual’s external environment (e.g., policy, culture, etc.) (4, 5). In social determinants of health theory, it is divided into five levels: individual behavior and lifestyle, social and community influences, and socioeconomic, cultural, and environmental (6). In social class differences theory, it argues that income level, socioeconomic status, occupational status, and education level determine health behaviors (7). Current research theories mainly focus on exploring the social capital on different groups of sports behavior research. There are few studies in the literature on the impact of individual cultural capital on physical activity.

Cultural capital is a form of capital that expresses residents’ cultural strengths or weaknesses, and is also an important mechanism for coping with social inequality and reducing social complexity (8, 9). According to Bourdieu, there are three forms of cultural capital that an individual can have (10, 11): First, embodied cultural capital. This form of cultural capital is the cultural product of upbringing, knowledge, skills and even literacy acquired through family and schooling. They are gradually accumulated through an individual’s cultural attitudes and social practices. The second is cultural capital in a physical state. This form of capital is physical objects that can be owned, such as books, tools, instruments, artifacts and other cultural wealth. Third, institutionalized cultural capital is the acquisition of academic qualifications and certificates through examinations. This is a sign that an individual’s knowledge, skills and abilities are recognized by society. Differences in the acquisition of the three forms of cultural capital among individuals lead to individual levels of cultural capital accumulation, which in turn shapes differences in residents’ knowledge levels, health awareness, and perceptions of exercise, affecting differences in residents’ choices to participate in physical activity (12, 13). Different levels of cultural capital shape differentiated physical and mental characteristics of individuals, and may also lead to the development of differentiated social behaviors, which in turn affects the individual’s perceptions, education, income, and even health (14). Previous studies have shown that family cultural capital had a significant positive effect on college students’ mental health (15).

Empirical research on how the three forms of cultural capital influence physical activity has focused on the separate associations of each form of capital (16). Theoretically, forms of capital interact with each other in multiple ways in their effects on health behaviors (17, 18). One form of capital is conditional on another if this form of capital can facilitate or restrict the use and acquisition of another form of capital (19, 20). For example, higher levels of literacy and education (institutional cultural capital) may result in more income (economic capital) providing opportunities to join in physical fitness behaviors or a willingness to purchase sports-related supplies (physical cultural capital), thus increasing individual physical fitness levels (21). In this example, the economic benefits of institutional cultural capital facilitated sport behavior choice. It is shown that cultural capital is an important driver of physical activity (22). By combing the concept of cultural capital, we know that inequality in the level of cultural capital may affect people’s participation in physical activity behaviors.

Previous studies have also demonstrated a correlation between institutional cultural capital (educational attainment), economic capital (household income), physical cultural capital (stock of sporting goods), and physical activity level (23). A high level of education raises individual economic incomes, with a consequent increase in socio-economic status (24–26). This facilitates individuals to optimize the allocation of resources for physical activity (27). Previous research has shown that these factors interact and complement each other. This study sorted out the relationship between cultural capital and residents’ participation in physical activity, innovatively explored the influence mechanism of the two, and provided a theoretical basis for the formulation of sports policies to improve the differences in residents’ physical fitness levels caused by class differences in physical fitness cognition and inequality in resource allocation brought about by cultural capital.

## 2 Methods

### 2.1 Data sources

Data in this study is from the 2020 China National Fitness Activity Status Survey among the Older Adult Population. This survey is a national census conducted every 5 years in Mainland China. Based on the principle of multi-stage stratified random sampling, a systematic probability sampling method proportional to the size of the population is used. 10–20 counties (county-level cities/districts/banners) were randomly selected in each of the 31 provinces (autonomous regions and municipalities directly under the central government) across the country then 13 villages (neighborhood committees) were randomly selected from each of the counties (county-level cities/districts/banners), and finally survey respondents were randomly selected from each of the villages (neighborhood committees). A total of 20,896 samples were obtained from 5,760 villages (neighborhood committees) in 471 counties through a questionnaire survey method combining household and electronic registration. All investigators or guardians signed an informed consent form before the formal investigation. The sample was sorted out to eliminate those that could not be used in this study, such as missing variables, invalid variables, and those that never participated in physical activity, and eventually a total of 17,154 valid samples were obtained. All the investigation protocols of this study received ethical approval from China Institute of Sport Science (CISS-2019-10-29).

### 2.2 Variable description

#### 2.2.1 Dependent variable

The purpose of this study is to analyze the effect of cultural capital on physical fitness level. Therefore physical fitness level is the dependent variable of this study. The questionnaire of the 2020 China National Fitness Activity Status Survey involves the frequency, time, and intensity of physical activity. Question D1 “How often did you participate in physical exercise in the past year?,” Question D2 “In most cases, how long do you work out each time?” and Question D3 “In most cases, what kind of physical feeling do you have each time you exercise?”. Based on those questions, the options were organized. The Physical Activity Rating Scale (PARS-3) (28, 47), which revised and finalized by Liang and Liu was used to calculate the physical fitness level score, including the three indicators of physical fitness intensity, physical fitness time, and physical fitness frequency. Physical Fitness Level = Physical Fitness Intensity Score × (Physical Fitness Time Score − 1) × Physical Fitness Frequency Score. All three aspects of intensity, time and frequency are divided into five levels, corresponding to scores of 1–5, with a maximum score of 100 for physical fitness level and a minimum score of 0.

#### 2.2.2 Independent variable

In this study, institutional cultural capital (educational level) and health capital (health status) are used as independent variables. Among them, institutional cultural capital refers to the level of personal education. In the questionnaire, the question is “what is your educational level?” (1) No schooling, (2) Literacy class, (3) Elementary school (including private school), (4) Junior high school, (5) High school/middle school/technical school, (6) College, (7) Bachelor’s degree, (8) Graduate school and above. The options were converted to (1) High school and below, (2) College and above. In the health status questionnaire, the question is “which of the following diseases do you have (confirmed by the hospital)?” The options included 27 common diseases such as diabetes, hypertension, hyperlipidemia, intervertebral disk disease, heart disease, stroke, and arthritis. The options are summed to assess the individual’s health status, with a higher number of co-morbidities indicating poorer health.

#### 2.2.3 Intermediary variable

The mediating variables are average annual household income (economic capital) and physical cultural capital (stock of sporting goods). Income was measured using the average annual household income in the questionnaire. Physical capital in this study refers to the stockpile of sports-related supplies or equipment, using the questionnaire, Has your household purchased sports and fitness equipment or sports gear? Basketball, volleyball, soccer, table tennis, badminton, tennis, dumbbells, fitness equipment (e.g., treadmill), skipping ropes, yoga balls, jumping ropes, bathing suits, professional running shoes. Sum the options to represent physical cultural capital.

#### 2.2.4 Control variables

The control variables included in this study are demographic variables commonly used in research: gender: 1 for female while 2 for male; age transformed into a continuous variable: 1 for 60–69 years old, while 2 for 70 and above; urban and rural: 1 for urban while 2 for rural.

### 2.3 Research hypothesis

**Figure 1:**
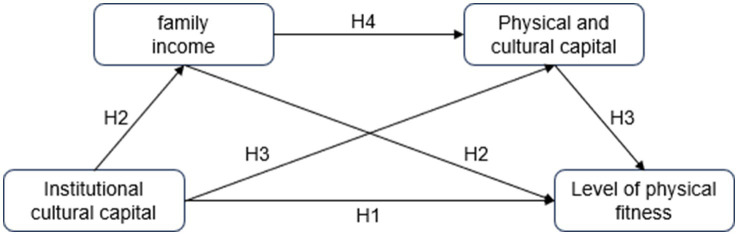
Research framework hypothesis.

Based on the first part of the analysis, we constructed the research framework in Figure 1 and put forward the research hypotheses of this study: H1: Institutional cultural capital has a positive impact on the level of physical fitness of the older adult; H2: Average household income plays a mediating role in the institutional cultural capital to improve the level of physical fitness of the older adult; H3: Physical cultural capital plays a mediating role; H4: Average household income and material cultural capital play a chain mediating role in institutional cultural capital to increase the level of physical fitness of the older adult (Figure 1).

### 2.4 Data analysis

The dependent variable in this study was assessed by the Physical Activity Rating Scale (PARS-3) to calculate the physical activity level score of the older adult population, and therefore, it was analyzed using stratified regression. In order to more clearly indicate the effects of the respective variables on the dependent variable, stratified linear regression was used and five regression models were constructed for analysis. Model 1 incorporated demographic control variables: age, gender, and urban/rural. Model 2 incorporated educational attainment (institutional cultural capital). Model 3 incorporated health status. Model 4 incorporates household income (economic capital). Model 5 incorporated stock of sport supplies or facilities (physical cultural capital). Further, we used quantile regression to explore the distribution of the effects of institutional cultural capital across physical fitness levels. The use of quantile regressions not only provided a robust test on the results of stratified linear regressions, but also allowed us to analyze the differential effects of institutional cultural capital among individuals with different fitness levels. Finally, we further explored the intrinsic pathways of sport participation on individual fitness levels using model 6 in PROCESS provided by Hayes et al. (48). Bootstrap methods were used to test the mediating effects of household income and physical cultural capital, setting a Bootstrap count of 5,000 and a confidence interval of 95%. All statistical procedures for this study were done in SPSS 27.0 and STATA 17.0.

## 3 Results

### 3.1 Descriptive statistics and correlation analysis

Table 1 showed the means, standard deviations and correlations of all variables. Pearson correlation coefficients showed that: among the control variables age, gender, urban and rural areas correlate significantly with the level of physical fitness in the older adult population (*p* < 0.01); among the key independent variables institutional cultural capital and individual health status correlate significantly and positively with the level of physical fitness (*p* < 0.01). In addition the mediating variable, there was a significant positive correlation between average household income and physical cultural capital (*p* < 0.01). Based on the results of correlation analysis, we will further verify the effect of institutional cultural capital and physical fitness level of the older adult (Table 1).

**Table 1:** Descriptive statistics and correlation analysis of variables.

	Physical fitness score	Age	Gender	Urban/rural	Institutional cultural capital	Health status	family income	Physical cultural capital
Physical fitness score	1							
							
Age	−0.058	1						
	0.000							
Gender	−0.017	0.002	1					
	0.512	1.000						
Urban/rural	−0.137	0.010	−0.023	1				
	0.000	0.000	0.000					
Institutional cultural capital	0.088	−0.148	−0.195	−0.299	1			
	0.000	0.000	0.000	0.000				
Health status	0.346	0.078	0.052	−0.051	−0.035	1		
	0.000	0.000	0.000	0.000	0.000			
family income	0.099	−0.025	−0.019	−0.286	0.397	0.002	1	
	0.000	0.036	0.311	0.000	0.000	1.000		
Physical cultural capital	0.120	−0.131	−0.022	−0.158	0.258	0.050	0.245	1
	0.000	0.000	0.000	0.000	0.000	0.000	0.000	
Mean	23.838	67.689	1.494	1.337	3.644	0.883	2.633	1.392
SD	18.240	6.464	0.500	0.473	1.349	1.231	1.787	1.894

Institutional cultural capital refers to academic qualifications, and physical cultural capital refers to stocks of physical fitness goods.

### 3.2 Chain regulation analysis of cultural capital and physical activity level

In model 1, age had a significant effect on the level of physical fitness of the older adult (*β* = −0.038, *p* < 0.01), and the level of physical fitness of people aged 70 years and above was low relative to that of people aged 60–69 years. Gender had a significant effect on the level of physical fitness among the older adult (*β* = −0.021, *p* < 0.01), with women having a lower level of physical fitness than men. Urban and rural areas also showed a significant effect (*β* = −0.137, *p* < 0.01) with the level of physical fitness of the older adult, with rural residents having a low level of physical fitness relative to urban residents.

Institutional cultural capital was included in Model 2, and there was a significant effect of institutional cultural capital with the level of physical fitness of the older adult (*β* = 0.051, *p* < 0.01), and the secondary school-educated population scored high in the level of participation in sports and fitness relative to the elementary school and below population. The physical fitness level of the college and above population was higher than that of the population with secondary school education (*β* = 0.051, *p* < 0.01).

Physical cultural capital was included in Model 3, and physical cultural capital was significantly affected with the level of physical fitness of the older adult (*β* = 0.089, *p* < 0.01), the higher the score of embodied cultural capital, the higher the level of participation in physical activity of the older adult population, and the embodied cultural capital mainly included common sense judgment of physical activity behavior.

The average annual household income was included in model 4, and the average annual household income had a significant effect on the physical fitness level of the older adult (*β* = 0.036, *p* < 0.01), and the higher the income the higher, the score of the physical fitness level of the older adult. The inclusion of average annual household income, the regression coefficient of institutional cultural capital changed significantly, the regression coefficient of the secondary school group decreased from 1.874 to 1.076, and the regression coefficient of the high school group decreased from 3.731 to 2.530.

The individual health status was included in Model 5, the higher the score of health status (negative indicator), the worse the health status. Health status is positively correlated to the level of physical fitness (*β* = 0.029, *p* < 0.01), the worse the health status the higher the level of physical fitness of the older adult (Table 2).

**Table 2:** Stratified linear regression analyses of cultural capital and physical fitness level.

		Model 1			Model 2			Model 3			Model 4			Model 5	
	*b*	SE	*β*	*b*	SE	*β*	*b*	SE	*β*	*b*	SE	*β*	*b*	SE	*β*
Age	−1.474	0.296	−0.038**	−1.194	0.299	−0.030**	−0.893	0.299	−0.023**	−0.930	0.299	−0.024**	−1.021	0.300	−0.026**
Gender	−0.750	0.276	−0.021**	−0.363	0.280	−0.010	−0.394	0.279	−0.011	−0.429	0.279	−0.012	−0.477	0.279	−0.013
Urban/rural	−5.291	0.292	−0.137**	−4.586	0.306	−0.119**	−4.263	0.306	−0.110**	−4.018	0.311	−0.104**	−3.954	0.311	−0.102**
Institutional cultural capital				1.874	0.303	0.051**	1.328	0.306	0.036**	1.076	0.311	0.030**	1.124	0.311	0.031**
				3.731	0.597	0.051**	2.530	0.604	0.034**	1.698	0.635	0.023**	1.742	0.635	0.024**
Physical cultural capital							0.854	0.075	0.089**	0.804	0.076	0.083**	1.124	0.311	0.031**
Family income										0.368	0.086	0.036**	1.742	0.635	0.024**
Health status													0.432	0.112	0.029**
*R*		0.021			0.024			0.031			0.032				0.033

(1) *p < 0.05, **p < 0.01. (2) Institutional cultural capital refers to academic qualifications, and physical cultural capital refers to stocks of physical fitness goods.

### 3.3 Quartile regression analysis of institutional cultural capital on physical fitness levels of older adults

In this section, we used quantile regression to further analyze the effect of institutional cultural capital on physical fitness levels of older adults. The quantile regression results showed that the impact of education level on physical fitness level showed an inverted U-shaped change in the regression coefficient from the low quartile 0.1 to the middle and high quartile 0.75, with the greatest impact on the quartile 0.5. The trend of the influence of sports equipment stockpile on physical fitness level gradually increases, and the influence of sports equipment stockpile on high fitness level is the largest, and the influence of family income on physical fitness level is consistent with the influence of education, which peaks at the middle and high quartile 0.75 and is not significant at the high quartile 0.90. The effect of health status on physical fitness level was not significant at 0.10 in the lower quartile and 0.90 in the higher quartile, again showing an inverted U-shape, peaking at 0.50 in the quartile. The factors favored the promotion of participation in moderate and higher levels of physical activity among older adults (Table 3).

**Table 3:** Effect of institutional cultural capital on physical fitness levels of older people: quantile regression analysis.

Independent variables	Dependent variable: level of physical fitness				
	0.10	0.25	0.50	0.75	0.90
Institutional cultural capital	0.625**	0.788**	1.059**	0.802**	−0.097
	0.000	0.000	0.000	0.000	0.626
Physical cultural capital	0.625**	0.973**	0.941**	0.967**	1.645**
	0.000	0.000	0.000	0.000	0.000
Family income	0.250**	0.398**	0.824**	1.120**	0.597
	0.000	0.000	0.000	0.000	0.141
Health status	−0.025	0.230*	0.824**	0.573**	0.290
	0.717	0.036	0.000	0.008	0.347
Control variables	YES	YES	YES	YES	YES
Observation	17,154	17,154	17,154	17,154	17,154

(1) *p < 0.05, **p < 0.01. (2) Health status is a negative indicator, with higher scores indicating a higher number of co-morbidities. (3) Control variables: age, gender, urban/rural.

### 3.4 Chain regulation analysis of cultural capital and physical activity levels

**Figure 2:**
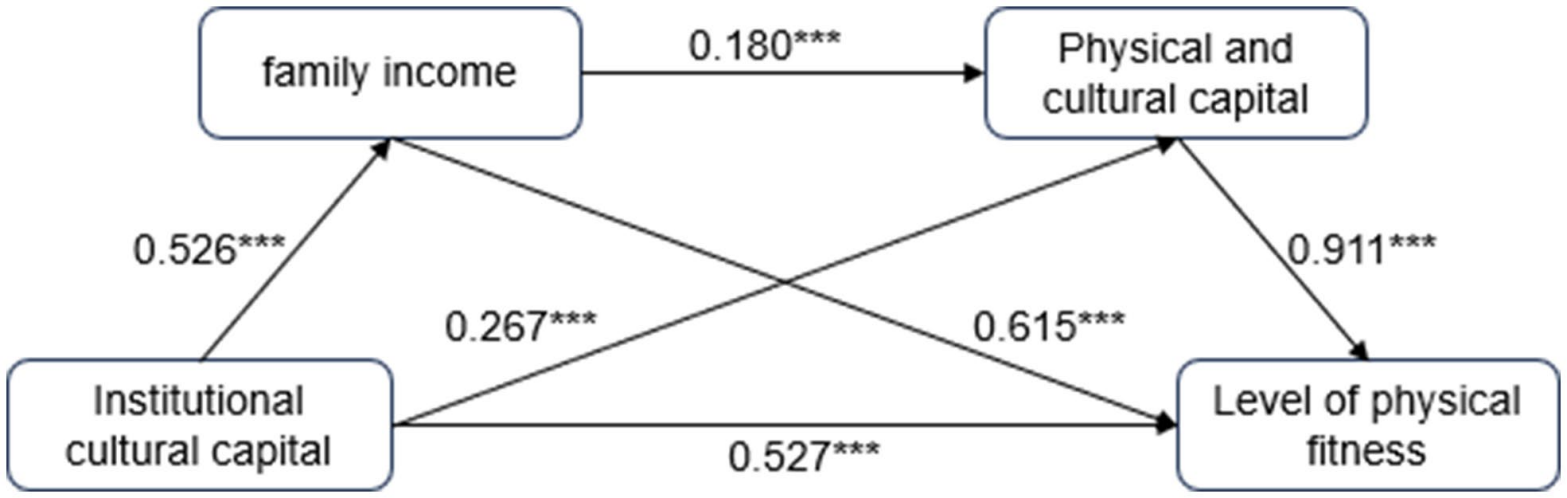
Chain mediation model of cultural capital and physical fitness level. ***p < 0.001.

Mediating effects analysis. In this section, we further investigated the mechanism of cultural capital and effects on physical fitness level of older people. In the theoretical analysis section, we elaborated the internal logic of cultural capital on physical fitness level from economic income and physical capital. In the correlation analysis, we have found that there was a significant correlation between cultural capital, economic income, physical capital, and physical fitness level. In the regression analysis, the coefficient of the influence of cultural capital on the level of physical fitness decreases after adding economic income and material capital variables. On this basis, this section explored the mechanism of the influence of cultural capital on physical fitness level. We constructed a chain mediating test model with physical fitness level as the dependent variable, cultural capital as the independent variable, and household income and material capital as the mediator variables, and the estimation results of the path coefficients are shown in Figure 2, and the results of the chain mediation effect analysis are shown in Table 4. In order to clearly demonstrate the interrelationships between the variables, we reported the standardized path coefficients in this section. We repeated the sampling 5,000 times using the Bootstrap method to analyze the main and chained mediation effects. The results showed that the path indirect effect with household income as the mediating variable is 0.323 (95% CI = [0.047, 0.235]), with a contribution of 49.49%; the path indirect effect with material capital as the mediating variable is 0.244 (95% CI = [0.199, 0.291]), with a contribution of 37.30%; and the path indirect effect with household income and material capital as mediating variables, an indirect effect of 0.086 (95% CI = [0.069, 0.105]) with a contribution rate of 13.22%. All indirect effects totaled 0.659 (95% CI = [0.554, 0.755]). The mediating effects of household economic income and physical capital in the influence of cultural capital on physical fitness levels were established (Figure 2; Table 4).

**Table 4:** Bootstrap analysis for significance test of mediation effect.

	Effect	BootSE	BootLLCI	BootULCI	Contribution rate
Total	0.653	0.052	0.554	0.755	
Institutional cultural capital—family income—physical fitness level	0.323	0.047	0.235	0.420	49.49%
Institutional cultural capital—physical cultural capital—physical fitness level	0.244	0.024	0.199	0.291	37.30%
Institutional cultural capital—family income—physical cultural capital—physical fitness level	0.086	0.009	0.069	0.105	13.22%

Institutional cultural capital refers to academic qualifications, and physical cultural capital refers to stocks of physical fitness goods.

## 4 Discussion

Using data from the 2020 China National Fitness Status Survey, we investigated the relationship between physical fitness level, institutional cultural capital, individual health status, average household income, and physical and cultural capital among the older adult population. Based on previous researches on the impact of cultural capital on physical activity behavior, we further explored the mechanisms by which cultural capital influences the physical activity level of older people. This study explored the influence of institutional cultural capital on the physical fitness level of the older adult population in China through five regression models, as well as mediation effects.

### 4.1 Cultural capital influences the level of physical fitness of older people

The formulation of cultural capital has a significant promotion effect on the improvement of physical fitness level of the older adult population, and the differences in cultural capital among individuals may lead to differences in physical fitness level. The results of the analysis show that the overall level of physical fitness of China’s older adult population is low, and each person suffers from one disease. The level of women’s participation in physical fitness activities is lower than that of men. As the older adult population grows older, the level of physical fitness declines. There are obvious economic differences between urban and rural areas in Chinese society, and the physical fitness level of the rural older adult population is also much lower than that of urban residents. This may depend on the fact that rural residents have a weak cognition of fitness, as well as limited physical fitness facilities due to economic development constraints. This is consistent with previous studies of the impact of demographic factors on sports participation in different populations (29–31).

Our results have suggested that institutional cultural capital, that is, educational attainment, promoted the level of physical fitness in the older adult population. That is, the higher the education level, the higher the level of physical fitness among older adults, possibly due to higher levels of education, more opportunities for sport participation, and a greater understanding of China’s promotion of the concept of fitness for all and a healthy China (32). Specifically, the effect of institutional cultural capital on the physical fitness level of older people exhibit a significant quantile effect, and the effect showed a significant linear upward trend as the quantile value increases. We further analyzed the pattern of the effect of sport participation on individual physical fitness levels. It may also be due to the emergence of social class separation in physical activity (33, 34). Numerous studies have confirmed that there are also significant differences in physical activity participation between urban and rural residents (35, 36) because urban residents have more access to physical fitness-related activities, such as sports clubs (37, 38). Institutional cultural capital represented by education and academic qualifications can improve the level of physical fitness of the older adult population, which reflected previous research: physical activity is related to a person’s socio-economic status, and educational attainment and academic qualifications symbolize the level of residents’ socio-economic status, with higher socio-economic status being more exposed to opportunities or resources for physical activity (39). Studies in Europe and the United States have also shown that people with higher education are also more physically active in their leisure time than their peers (40).

### 4.2 Household income and stockpiles of sporting goods have mediating effect

The analysis in the mechanism of the influence of cultural capital on the level of physical fitness of the older adult shows that institutional cultural capital enhances the level of physical fitness of the older adult population through economic income and physical cultural capital reserves. Economic income and physical cultural capital play a significant chain mediating role. We focused on exploring the intrinsic links between them according to the theoretical framework. Institutional cultural capital largely raises average annual household income and symbolizes individual economic status. Good cultural capital also represents higher cultural education and positive judgment of physical fitness value. Moreover, a good household income can encourage individuals to stock up on sports-related supplies, which greatly improves the convenience of physical fitness. The stockpiling of sports-related supplies also symbolizes the individual’s interest preference, which has a positive effect on older adults’ sports behavior (41). Our research hypothesis was validated by the fact that high levels of education led to high economic income, which promotes individual stockpiles of sporting goods, thereby increasing physical fitness levels in the older population. Income reward promotion theory suggests that higher levels of education mean better professional and financial income. Beneficial to individuals’ ability to access sports resources (39). Previous studies have also demonstrated that high household income favors the participation of children and adolescents in more physical activities and can support their purchase of sport-related products (42, 43).

The educational level of the population plays an important role in the cultural capital as a whole, which is only a natural form of social difference, but it’s a social phenomenon produced through the hierarchical and differentiated cultural capital in the dominant relationship (44). The perceived value of sport, knowledge of health and even differences in socio-economic status that it brings about can be improved and optimized through national policies (45, 46). From the viewpoint of institutional cultural capital, differences in economic income, differences in cognitive level, and unequal distribution of sports resources caused by differences in the educational level of China’s older adult population, thus affecting the level of physical fitness of the older adult population. From the perspective of material cultural capital, the accessibility of sports equipment for the older adult in China relies on family financial support or community sports resource provision (32). Good cultural capital is something that takes a lot of time and effort to accumulate, so there is some variation in residents’ cultural capital, which affects their physical activity. This also explained why cultural capital can explain the logic inherent in physical activity participation.

### 4.3 Research limitations

There are some limitations in this study. For example, the cross-sectional data used in this study did not allow for the exploration of temporal relationships between variables. Therefore, longitudinal tracking can be included in subsequent studies. In addition, cultural capital is divided into embodied cultural capital, material cultural capital, and institutional cultural capital. Also, this study only elaborated on institutional cultural capital and material cultural capital, which can be combined with the three forms of cultural capital to explore the relationship with individual sports behaviors in future studies.

## 5 Conclusion

This study innovatively investigated the mechanism of the influence of cultural capital on the physical fitness level of the older adult population, and found that economic income and the stockpiling of sports equipment play a mediating role in the influence of education on the physical fitness level of the older adult. Therefore, it is recommended that: Focus on the role of cultural capital in promoting physical fitness level at the same time as fitness promotion, increase the promotion of sports values in cultural education, and improve the residents’ perception of sports values. Focus on public health service training for grassroots, so that residents can enjoy advice and guidance on health equity and sports-related knowledge, and make up for the differences in class perception caused by cultural capital. Encourage residents to stockpile sports and fitness equipment or improve community sports facilities to improve the convenience of fitness and accessibility of sports resources.

Future research, such as longitudinal studies, can be conducted to determine the causal logic relationship between variables in order to promote physical activity among the older adult population and achieve healthy aging. Additionally, studies could be conducted with different populations, such as children, adolescents, and adults, to explore the impact of different forms of cultural capital on fitness activities across the whole lifespan.

## Data Availability

Publicly available datasets were analyzed in this study. Data in this study is from the 2020 China National Fitness Activity Status Survey among the Elderly Population: https://www.sport.gov.cn/n315/n329/c24335053/content.html.
